# *Salmonella enterica* Growth Conditions Influence Lettuce Leaf Internalization

**DOI:** 10.3389/fmicb.2019.00639

**Published:** 2019-04-18

**Authors:** Yulia Kroupitski, Rachel Gollop, Eduard Belausov, Riky Pinto, Shlomo Sela (Saldinger)

**Affiliations:** ^1^Microbial Food-Safety Research Unit, Department of Food Science, Agricultural Research Organization, The Volcani Center, Rishon LeZion, Israel; ^2^Confocal Microscopy Unit, Institute of Plant Sciences, Agricultural Research Organization, The Volcani Center, Rishon LeZion, Israel

**Keywords:** *Salmonella*, internalization, stomata, attachment, leaf, lettuce, stress, *E. coli*

## Abstract

Human pathogens on plants (HPOP) have evolved complex interactions with their plant host. Stomatal internalization is one such mode of interaction, where bacteria are attracted to stomata and penetrate into the substomatal cavity by a process mediated by chemotaxis. Internalization enables HPOP to evade the hostile environment of the leaf surface and find a protected, nutrient-rich niche within the leaf. Numerous studies have documented attachment and entry of the foodborne pathogens, *Salmonella enterica* and *Escherichia coli* into stomata. Internalization, however, varies considerably among different pathogens and in different plants, and both bacterial and plant’s factors were reported to influence HPOP attachment and internalization. Here we have studied the effect of laboratory growth conditions, on the internalization of *Salmonella enterica* serovar Typhimurium (STm) into lettuce leaf. We have further tested the potential involvement of universal stress-proteins in leaf internalization. We found that STm grown in Luria Bertani broth devoid of NaCl (LBNS), or in diluted LB (0.5×LB) internalized lettuce leaf better (62 ± 5% and 59 ± 7%, respectively) compared to bacteria grown in LB (15 ± 7%). Growth under non-aerated conditions also enhanced STm internalization compared to growth under aerated conditions. Growth temperature of 25 and 37°C did not affect STm internalization, however, growth at 42°C, significantly augmented leaf internalization. Since, the tested growth conditions represent moderate stresses, we further investigated the involvement of five universal-stress genes in STm leaf internalization following growth in LBNS medium. Knockout mutations in *ydaA, yecG, ybdQ*, and *uspAB*, but not in *ynaF*, significantly reduced STm internalization compared to the wild-type (wt) strain, without affecting bacterial attachment and motility. Transduction of the mutations back to the parent strain confirmed the linkage between the mutations and the internalization phenotype. These findings support a specific role of the universal-stress genes in leaf internalization. The present study highlights the complexity of bacterial internalization process and may provide partial explanation for the variable, sometimes-contrasting results reported in the literature regarding stomatal internalization by HPOP. Characterization of the regulatory networks that mediate the involvement of *usp* genes and the tested growth factors in STm internalization should contribute to our understanding of human pathogens-plant interactions.

## Introduction

Consumption of fresh vegetables has consistently increased over the last decades, since they are considered an important part of a healthy and low-calorie diet (Tirpanalan et al., 2011). Concomitantly, there is an increase in outbreaks of food-borne illness associated with the consumption of fresh produce (Brandl and Sundin, 2013; Mritunjay and Kumar, 2015). Lettuce is one of the vegetables, which often linked to outbreaks of foodborne diseases. For example, from 1994 to 2011, more than 20 alerts associated with contaminated lettuce were reported through the RASFF portal of the EU (Tirpanalan et al., 2011). Although efforts to develop chemical and technological means to disinfect vegetables are ongoing (Meireles et al., 2016), there is currently no treatment that can effectively kill foodborne pathogens on fresh produce (Goodburn and Wallace, 2013).

Numerous studies have documented the ability of foodborne pathogens, such as *Escherichia coli* and *Salmonella enterica*, to colonize plants and interact with the plant immune system (Schikora et al., 2012; Garcia and Hirt, 2014) and are considered human pathogens on plants (HPOP). Several reports have demonstrated that HPOP may enter the plant tissues through roots or via natural openings on the plant surface, such as stomata, lenticels or sites of biological or physical injury (Itoh et al., 1998; Seo and Frank, 1999; Takeuchi and Frank, 2001; Solomon et al., 2002; Kroupitski et al., 2009; Deering et al., 2012; Martínez-Vaz et al., 2014). Internalization enables HPOP to evade the hostile environment of the soil and the plant surfaces and find a protected, water- and nutrient-rich niche within the plant. Internalization may also protect internalized HPOP against surface sanitation used by the fresh produce industry (Gil et al., 2009; Niemira and Cooke, 2010). Consequently, understanding the factors that affect plant internalization by HPOP is needed toward developing new approaches to enhance the safety of fresh produce.

Attachment and internalization of HPOP are complex processes depending on both bacterial and plant factors. Plant’s factors include, plant growing conditions (Ailes et al., 2008; Ge et al., 2012; Lopez-Galvez et al., 2018), development stage (Brandl and Amundson, 2008; Kroupitski et al., 2009; Pu et al., 2009), plant’s cultivar (Jablasone et al., 2005; Mitra et al., 2009; Barak et al., 2011; Quilliam et al., 2012), as well as contamination site (Hirneisen et al., 2012). Bacterial factors that affect attachment and internalization include, cell concentration (Pu et al., 2009; Erickson et al., 2010; Ge et al., 2012), bacteria strain (Klerks et al., 2007; Wright et al., 2013; Kljujev et al., 2018) and biofilm formation (Yaron and Römling, 2014).

It has been demonstrated that preadaptation of human pathogens, including *Salmonella enterica*, to mild stresses enhanced their survival under more extreme stresses, as well as under other stress, in a process termed, cross protection (Jenkins et al., 1998; Bunning et al., 1990; Chung et al., 2006; Yuk and Schneider, 2006; Alvarez-Ordóñez et al., 2015). It has been reported that exposure of bacteria to the vegetable environment stimulates a cellular response required to colonize the animal host intestine (Goudeau et al., 2013). More recently, Fornefeld et al. (2017) have demonstrated that pregrowth of *Salmonella* Typhimurium LT2 in lettuce medium, rather than in rich laboratory broth (Luria Bertani, LB) resulted in better survival of the pathogen in soil microcosms. It has been suggested that the medium used to pregrow *Salmonella* can influence its fate in the environment (Fornefeld et al., 2017). Likewise, we hypothesize that pregrowth conditions of laboratory-grown *S.* Typhimurium may also affect bacterial response required for interaction with the plant host, and specifically, affect stomatal internalization using lettuce leaf model system.

## Materials and Methods

### Bacterial Strains, Inoculum Preparation, and Fluorescence Labeling

*Salmonella enterica* serovar Typhimurium (STm) SL1344 strains and *E. coli* O157:H7 EDL933 strain were labeled with mCherry-fluorescence protein (mCherry) by electroporating plasmid pKB2690, containing the mCherry gene and ampicillin resistance gene (Sason et al., 2009). Bacterial cultures were kept in Luria-Bertani (LB; 10 g Bacto-peptone, 5 g Yeast Extract, 10 g NaCl) broth containing 25% glycerol at -80°C. For each experiment, bacteria were streaked on LB agar for overnight and fresh colony were re-suspended in 10 ml LB broth and grown with shaking (150 rpm) for 18–20 h at 37°C to generate the inoculum for the internalization assay. In some cases, as indicated, bacteria were grown at 25 and 42°C. Other growth media that were used, included LB broth without NaCl (LBNS), and water-diluted LB broth (0.5×LB). Where indicate, bacteria were grown in LB broth without shaking or on LB agar plates. Overnight liquid cultures were washed twice with sterile saline (0.85% NaCl) by centrifugation (2700 g, 10 min) and re-suspended in saline to a final concentration of about 10^8^ colony-forming units (CFU) per ml.

### Generation of Knockout Mutants in Universal Stress Genes

Site-directed mutagenesis was performed as described by Datsenko and Wanner (2000) using primers specific to each of the mutated genes (Table 1). The absence of the intact gene in the mutants and the authenticity of the nearby DNA sequences were confirmed by PCR and sequence analyses using upstream- and downstream-chromosomal derived primers in combination with the respective Km-cassette derived primer. A list of primers used to generate the mutants and to confirm their sequence is present in Table 1.

**Table 1:** Primers used in this study.

			References/
Primer	Forward sequence	Reverse sequence	source
uspAB	TCATGAGCGCAATCAAGCTCACCACCACCAAACCG CATAACGCGCTGGTCTGTAGGCTGGAGCTGCTTCG	GCAGCGGCACAATCAGCATGTCAACGTGAACGGTG TTGATCAGCTGGCGCCATATGAATATCCTCCTTAG	This study
uspA-RT	ACGTCAATCTGGGCGATATG	GCTCAGGGTTTCAGTGATAGG	This study
yecG	TCTATCGCCCGCCCTGTTCAGGCGAAAGTAAGCCT GATTACTCTCGCTTCTGTAGGCTGGAGCTGCTTCG	TGCCGAGCAGGACGCGCGCGAAAAGAAACTGTGG TTATGGTTGCCGCAAACATATGAATATCCTCCTTAG	This study
yecG-RT	AGGATTTACGCGCGGTTATG	ACTCACCTGATGCGATGAAAG	This study
ybdQ	GCGCAACAGGATGGCGTCATTCATCTGTTGCATGTA CTGCCTGGGTCCGCGCATATGAATATCCTCCTTAG	ACCACGCTTGAGGCGTTAGACCCCAACAGGTGTGT CGTGATGGACGGATTTGTAGGCTGGAGCTGCTTCG	This study
ybdQ-RT	AAACGATGGTGGGACACTTC	GACATCCGCATCCAGTTCTT	This study
ynaF	CCCATCGATATTTCAGATTCAGAATTAACTCAACGC GTGATTTCGCATGTTGTAGGCTGGAGCTGCTTCG	GAGCATTCCGCATGACGCACAACGGCTGCGGCGT TGGAACCCAACAGATACATATGAATATCCTCCTTAG	This study
ynaF-RT	CTTCACTGGGACTGGCTTATT	GCGGGAAGGTTGAATTTCTTG	This study
ydaA	CTGCGATGACAGTTGTAAGGAGACCCTGTATGGCT ATGTATCAAAATATGTGTAGGCTGGAGCTGCTTCG	CAGTTCAACCGGTGTTTGATACTCATCAGGCTTAA TGACTAACAGGTCGCCATATGAATATCCTCCTTAG	This study
ydaA-RT	CCGTGTGGATGGTCAAAGAT	TCGTTGAGAGCATTGTGATAGG	This study
rpoD-RT	GGCTCGTTTGTCCGATCTTAT	CTTCGTCATCATCCAGGTCTTC	This study
k2	CGGTGCCCTGAATGAACTGC		Datsenko and Wanner, 2000
kt		CGGCCACAGTCGATGAATCC	Datsenko and Wanner, 2000
*uspAB*test	GAAACTGGCCCGCTTTTT	TATAGACCAGACGCGGTCTTAGC	This study
*yecG*test	GCACAATCTCATATTCTTGCAATC	GTGGCGACGTTCCCTAAG	This study
ybdQtest	CGGTTGATGTTTTTGAAATGG	GGCAGGGCGTGTAAGTTTT	This study
ynaFtest	AGTATCGTGGAGCAGCACCT	GGCGATGATGATTGATTTGA	This study
			This study
ydaAtest	GCGCTTCCTCTGTTTCATTC	AATAGGGTATTGGCCGGATG	This study

To confirm the linkage between the leaf internalization phenotype and the *usp* mutations, each of the mutation was further transferred to the STm wt strain by P22 HT int-105 transduction, as described (Kroupitski et al., 2013). Transductants were isolated on Km plates and the presence of the specific mutation was confirmed by PCR using primers presented in Table 1.

### Reverse Transcription Real Time PCR

STm SL1344 strain was grown in various media and growth conditions, as described above. Three milliliters of an overnight cultures (about 10^9^ cells) were harvested by centrifugation (2700 g, 10 min) and total RNA was isolated by the RNAqueous^^®^^ kit (Ca. No. AM1912), according to the manufacturer instructions (Ambion). DNase treatment and cDNA synthesis were performed using Maxima first strand cDNA synthesis kit with dsDNase (Thermo Scientific; Ca No. K16171). Primers for real time PCR of *uspA, yecG, ybdQ, ydaA, ynaF*, and *rpoD* genes were selected from STm SL1344 genomic sequence using the Syntezza-Israel IDT web portal (Integrated DNA Technologies; Leuven, Belgium), through the primer quest tool and are listed in Table 1. Fast SYBR^^®^^ Green Master mix (AB#4385612), 96-wells plates (AB#4346906) and adhesive covers (AB#4311971) were purchased from Applied Biosystems. Real time PCR reactions (10 μl), included 1.72 ng cDNA, 0.2 μM each forward and reverse primers and 50% volume of Fast SYBR^^®^^ Green Master mix (Applied Biosystems). Transcription of *uspA, yecG, ybdQ, ydaA*, and *ynaF* genes was evaluated by Real time PCR using Applied Biosystems StepOnePlus^TM^ PCR system (Applied Biosystems, Foster City, CA, United States). The PCR conditions comprised 20 s at 95°C followed by 40 cycles at 95°C 3 s and 60°C for 30 s. To verify the specificity of each primer, a melting-curve analysis was included (60–95°C with fluorescence measured every 0.3°C). The threshold cycle (C*_T_*) value and relative quantification (RQ) level were determined by StepOne^TM^ Software 2.1 (Applied Biosystem). The house-keeping gene, *rpoD* (Kjeldgaard et al., 2011) served as a reference gene for normalization. Each run included a negative control and a cDNA reaction without reverse transcriptase to rule out DNA contamination. All experiments were performed in triplicates.

### Lettuce Preparation

Fresh whole iceberg lettuce (*Lactuca sativa*) was purchased at a local retail store, and used immediately or kept in a refrigerator for 1 day. Before each experiment, the lettuce temperature was equilibrated at room temperature. The outermost leaves of the lettuce head were aseptically removed and the inner 2–3 leaves were taken for the experiments. The leaves were aseptically cut into ca. 3 × 3 cm pieces using a sterile scalpel, as described previously (Kroupitski et al., 2009).

### Leaf Attachment and Internalization Assays

Interaction of bacteria with leaf pieces were tested, as described before (Kroupitski et al., 2009). Briefly, lettuce pieces were submerged in 30 ml saline in a 50 ml sterile polypropylene tube (Labcon, Petaluma, CA, United States), at one piece per tube. The leaves were pre-conditioned for 20 min under high intensity light (100 μE m^-2^ s^-1^) at 30°C. The saline was then replaced with 10 ml of mCherry-labeled bacterial suspension (in saline) containing about 10^8^ CFU per ml. The tubes were incubated for 2 h under the same conditions. Leaf pieces were rinsed twice for 1 min in excess of saline to remove unattached bacteria and an internal small piece (10 × 5 mm) was excised and viewed immediately under confocal laser-scanning microscopy (CLSM). Bacterial localization was determined on the leaf surface (attachment) and in deeper layers (internalization) in 30 randomly chosen microscopic fields (magnification ×40). Internalization was quantified as the incidence of internalized bacteria, i.e., percentage of fields (×40) containing ≥1 internal mCherry-tagged bacteria in 30 microscopic fields of the same leaf tissue. STm attachment was scored as the number of microscopic fields containing: 0, 1–10, 10–50, 50–100, and >100 fluorescent cells per 90 fields (×40). Each experiment included three lettuce pieces from different leaves (in total 3 × 30 microscopic fields were examined per experiment), and was repeated independently at least three times at different days, with different lettuce heads.

### Confocal Laser-Scanning Microscopy

mCherry-fluorescent bacteria were visualized by CLSM (Olympus IX81, Tokyo, Japan), using 40 × 0.7 objective. Fluorescence bacteria were visualized using excitation wavelength of 543 nm and a BA560-600 nm emission filter. Chlorophyll autofluorescence was detected using 488 nm excitation wavelength and emission filter BA 660 IF. Transmitted light images were obtained using Nomarski differential interference contrast (DIC).

### Motility Tests

Swarming and Swimming motility assays were performed, as described previously with minor changes (Kjeldgaard et al., 2011). Briefly, freshly grown bacterial cultures were suspended in saline to a final concentration of ca. 10^8^ CFU/ml. For swarming motility assay, bacterial suspensions (1 μl) were inoculated on the center of a swarm plate containing nutrient broth (NB) supplemented with 0.5% glucose and 0.6% agar. For testing swimming motility, a similar inoculum was inoculated with a sterile needle in the center of a well, in 12-wells plates, containing NB-glucose with 0.3% agar. The plates were incubated at 30°C for 18 h in a humid chamber. Swarming motility was assessed by measuring the radius of the swarm from the point of inoculation. Swimming motility was determined visually by the presence of turbidity in the entire volume of the inoculated well. The experiments were repeated independently five times. Nonmotile STm mutants, *fliGHI*, and motA (Kroupitski et al., 2009), served as an internal negative control for both motility assays. Swarming motility was presented as the average radius of the colony from the point of inoculation and standard errors of the means.

### Statistical Methods

Comparison of the incidence of bacterial attachment and internalization was performed by ANOVA using the program Instat, version 3.0.6 (GraphPad Software, Inc., La Jolla, CA, United States). The comparisons of the relative quantity of the amplified mRNA in the reverse transcription real-time PCR experiments were done by unpaired Student’s *t*-test using GraphPad software. Statistical significance was set at a one tailed *P* ≤ 0.05.

## Results

### Effect of Growth Conditions on *Salmonella* Internalization

**Figure 1:**
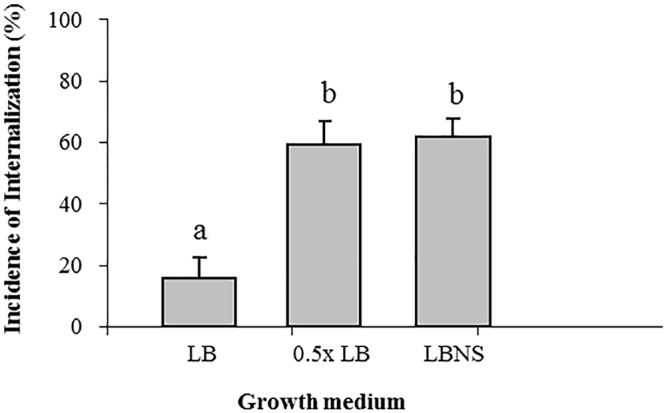
Effect of growth medium on the internalization of *Salmonella* in lettuce leaf. *Salmonella* was grown overnight in the following media: LB, LBNS, water-diluted LB (1:1) with shaking at 37°C, washed and tested for leaf internalization. Incidence of bacteria in internal leaf tissues was calculated as the percentage of microscopic fields (×40) harboring ≥1 mCherry labeled bacteria in 30 randomly chosen microscopic fields of leaf tissue. The data denote the average and standard error (SE) of four independent experiments each performed in triplicates (3 × 30 fields per experiment). Different letters indicate significant difference (*P* < 0.05) between means of internal fields harboring bacteria, according to ANOVA Tukey–Kramer Multiple Comparisons Test.

Growth of STm in LB broth resulted in the lowest incidence of internalization (15% ± 7), while growth in LBNS, or diluted LB (0.5×LB) resulted in a fourfold higher incidence of internalization, 62% ± 5 and 59% ± 7, respectively (Figure 1).

**Figure 2:**
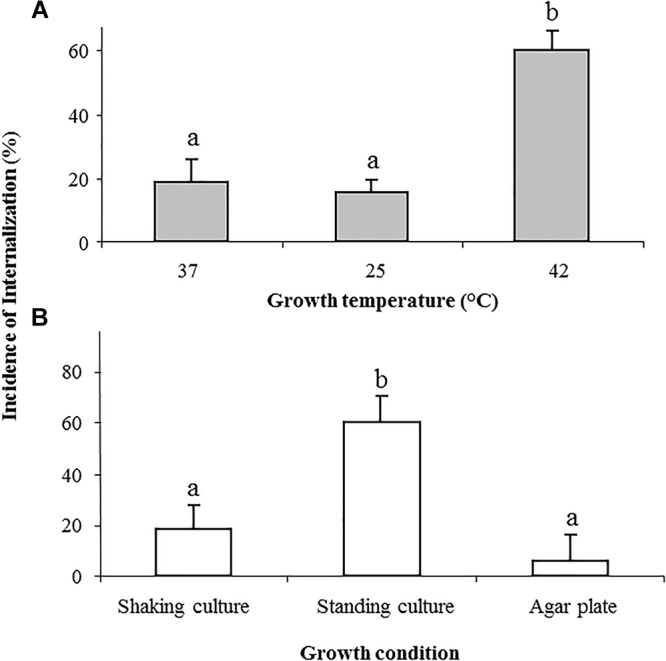
Effect of growth temperature and growth conditions on the internalization of *Salmonella* in lettuce leaf. *Salmonella* was grown overnight in LB broth at the following temperatures: 37, 25, and 42°C with shaking **(A)**, or in LB without shaking, as well as in LB plates, both at 37°C **(B)**. Cells were washed and tested for internalization. The incidence of internalization was calculated and is presented as described in the legend of Figure 1. The data denote the average and SE of four independent experiments each performed in triplicates. Different letters indicate significant difference (*P* < 0.05) between means of internal fields harboring bacteria, according to ANOVA Tukey–Kramer Multiple Comparisons Test.

Growth temperature of 25 or 37C yielded similar incidence of *Salmonella* internalization (19% ± 6 and 16% ± 3, respectively), however, growth at 42°C significantly increased bacterial internalization (60% ± 6; Figure 2A).

Growth of STm in LB broth at 37°C, without shaking, also enhanced internalization (61% ± 11) compared to growth in shaking culture (18 ± 6%) (Figure 2B). On the other hand, bacteria that were grown on LB agar were poorly internalized (6 ± 1%) (Figure 2B).

### Effect of LBNS on *E. coli* Internalization

**Figure 3:**
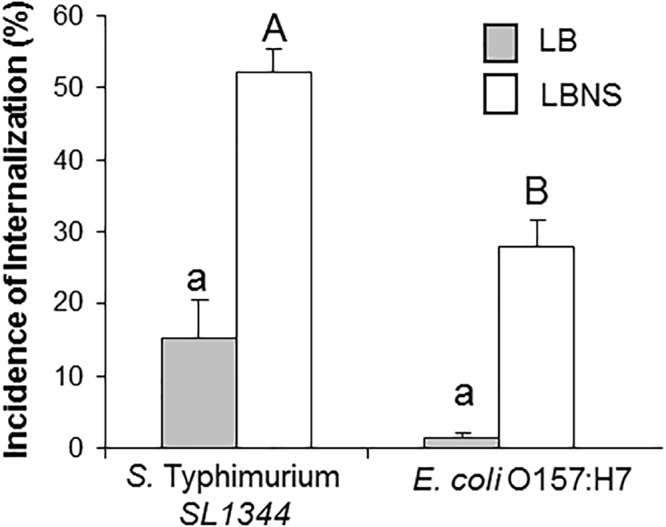
Internalization of *E. coli* in lettuce leaf. *Salmonella* SL1344, and *E. coli* O157:H7 were grown overnight in LB or LBNS medium, washed and tested for internalization. The incidence of internalization was calculated and is presented as described in the legend of Figure 1. The data are the average of 4 independent experiments each performed in triplicates. Different letters indicate significant difference (*P* < 0.05) according to ANOVA Tukey–Kramer Multiple Comparisons Test.

Previous studies in our laboratory have failed to show substantial leaf internalization in the case of *E. coli* O157:H7 EDL933 pregrown in LB broth (data not shown). Given our findings regarding the effect of growth medium composition on STm internalization, it was of interest to examine, whether this is a more general phenomenon. Therefore, leaf internalization of *E. coli* O157:H7 pregrown in either LB broth or in LBNS was also tested. Similar to *Salmonella*, pregrowth of *E. coli* strains in LBNS broth resulted in higher leaf internalization compared to growth in LB broth (28 ± 3% and 1.8 ± 1.2%, respectively) (Figure 3).

**Figure 4:**
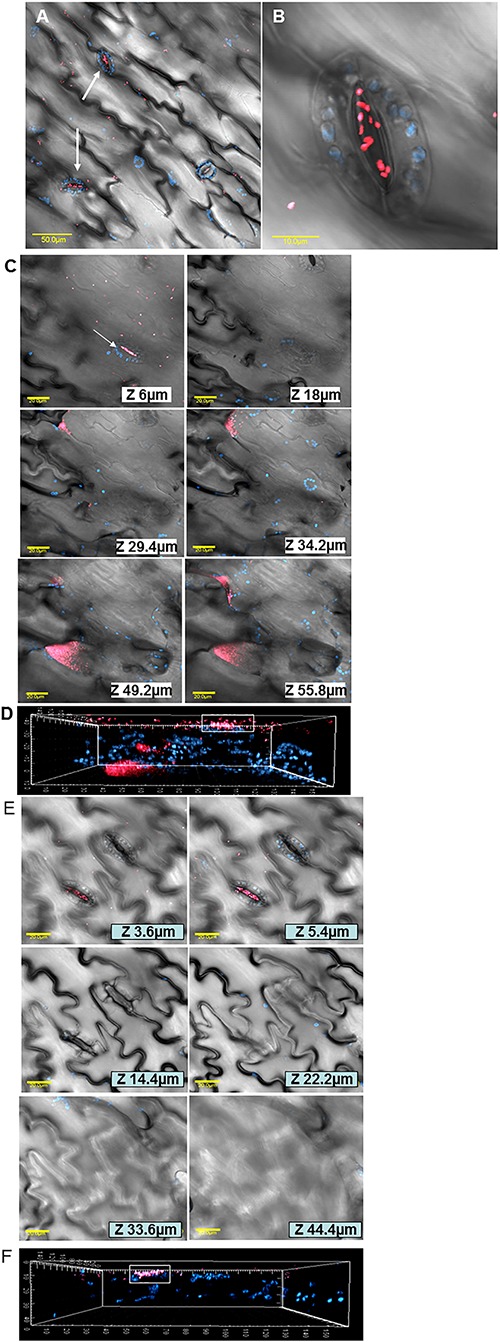
Microscopic analysis of *E. coli* O157:H7 interactions with lettuce leaf following growth in LB or in LBNS. Confocal microscopy images of mCherry-tagged *E. coli* (pink) grown in LB broth show both diffuse attachment to the leaf surface and stomata-clustering **(A)**. A higher magnification of a single stoma harboring *E. coli* cells is also presented **(B)**. Representative photomicrographs images showing LB-grown bacteria in various depth underneath the leaf surface **(C)** and a three-dimensional reconstruction of fluorescent images taken at the same leaf section, shown above, demonstrates the existence of bacteria (pink) in deeper leaf tissues **(D)**. In contrast, to LB-grown bacteria, following growth in LBNS, *E. coli* cells are detected in stomata but apparently no cell is observed within the leaf tissue **(E)**. Indeed, a three-dimensional reconstruction of confocal microscopy images taken at the same leaf section shown in **(E)**, shows no bacterial cells in the inner leaf tissues **(F)**. Blue fluorescence indicates auto-fluorescence of chlorophyll within chloroplasts of guard-cells and parenchymal cells. The fluorescent images in **(A–C,E)** were overlaid with the transmitted light image obtained using Nomarski differential interference.

In order to document the localization of LB- and LBNS-grown *E. coli* cells within the leaf tissue, mCherry-labeled bacteria were visualized by confocal microscopy. LBNS-grown bacteria were observed attached to the leaf surface with a distinct clustering pattern near and within stomata (Figure 4A,B). Images taken at various depths underneath the leaf surface demonstrated the presence of tagged bacteria within stomata and in the intercellular space (apoplast) of the spongy parenchyma in up to 55 μm depth (Figure 4C). A three-dimensional reconstruction model of fluorescent images taken at the same leaf region shows the presence of *E. coli* cells (pink) within a stomate (shown by the white rectangle) as well as in deeper layers of the leaf (Figure 4D). Similar z-section images and 3D reconstruction model performed on fluorescent images taken from leaves interacted with LB-grown *E. coli*, show no bacterial cells underneath the leaf surface (Figure 4E,F).

### Involvement of *Salmonella* Universal Stress Proteins in Leaf Internalization

**Figure 5:**
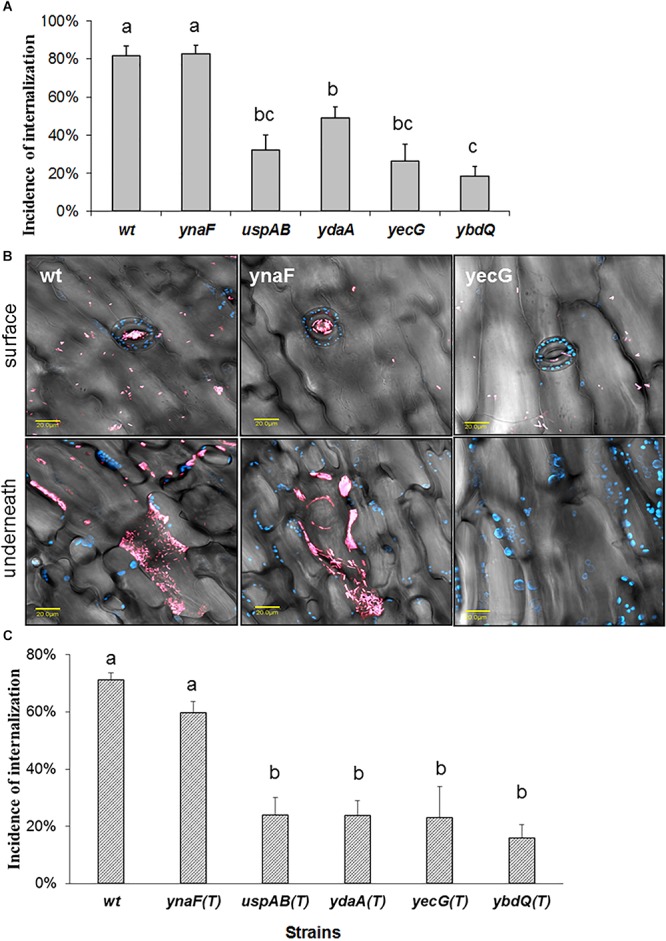
Effect of mutations in genes encoding universal stress proteins on *Salmonella* internalization. *Salmonella* wt and mutant strains were grown in LBNS and tested for internalization as described in Figure 1. The incidence internalization in lettuce leaf is presented **(A)**. Representative confocal microscopy image taken on the leaf surface, or stacks of images taken along a z-section underneath (inside), show *Salmonella* distribution on the surface and within the leaf tissue **(B)**. Blue color indicates auto-fluorescence of chloroplasts and pink color represent mCherry-tagged cells. The fluorescent images were overlaid with the transmitted light image obtained using Nomarski differential interference. The incidence of internalization of transductants harboring mutations in usp genes is presented in **(C)**. The data presented in **(A,C)** are the average and SE of 6 independent experiments each performed in triplicates (3 × 30 fields per experiment). Different letters indicate significant difference (*P* < 0.05) according to ANOVA Tukey–Kramer Multiple Comparisons Test.

The extrinsic factors that enhanced STm internalization, included mild stresses, such as growth in low NaCl broth (LBNS), nutrients deficiency (diluted LB broth), high-temperature (42°C), and low-oxygen content (standing culture without shaking). *Salmonella* possess at least five genes (*ydaA, yecG, ybdQ, ynaF*, and *uspAB*), encoding universal stress proteins, whose function in *Salmonella*-plant interaction is not known. To study possible involvement of these genes in *Salmonella* internalization, mutations in *ydaA, yecG, ybdQ, ynaF*, and *uspAB* were generated in STm SL1344. The wild type (wt) strain and the five isogenic mutants were grown in LBNS broth, and tested for leaf internalization. Mutants *uspAB, ydaA, yecG, ybdQ*, but not *ynaF*, demonstrated significant reduction in internalization efficiency compared to the wt strain (Figure 5A). Representative confocal microscopy images illustrating the localization of wt STm and two mutant strains on the leaf surface and underneath the surface are presented in Figure 5B). To confirm that the phenotype of the mutants was linked to the presence of the specific knockout mutations, each of the mutations was transferred back to the wt strain by transduction. The transductants harboring mutations in *uspAB, ydaA, yecG*, and *ybdQ*, but not in *ynaF*, were also impaired in leaf internalization phenotype, similar to the original knockout mutants (Figure 5C). These findings provide further evidence regarding the role these genes have in STm leaf internalization.

The wt and *ynaF* mutant strains display substantial attraction to stomata and were located in high numbers within the leaf tissue. In contrast, only few cells of the *yecG* mutant strain were observed on the leaf surface, including stomata, while no cells were observed underneath the surface Figure 5B).

It is possible that the low internalization phenotype displayed by the mutants merely reflect impaired attachment to the lettuce leaf, or lack of motility, which is required for leaf internalization (Kroupitski et al., 2009). Confocal microscopy visualization demonstrated that both the wt and its isogenic mutants displayed comparable attachment to the leaf surface (Table 2). Furthermore, both swarming motility (Table 3) and swimming motility (data not shown) of all the mutants were similar to that of the wt strain, suggesting that *uspAB, ydaA, yecG*, and *ybdQ* genes have a specific role in leaf internalization, which does not involve motility or attachment.

**Table 2:** Attachment of STm wild-type and mutant strains to lettuce leaf.

	Number of microscope fields out of 90 (means ± SD)				
	harboring mCherry-tagged STm cells				
Strains	0	1–10	10–50	50–100	100 ≤
wt	0	4 ± 3	53 ± 11	23 ± 6	8 ± 8
Δ*ynaF*	0	1 ± 0.5	47 ± 14	28 ± 11	13 ± 8
Δ*uspAB*	0	16 ± 7	47 ± 10	16 ± 6	6 ± 4
Δ*ydaA*	0	5 ± 3	57 ± 11	17 ± 6	10 ± 10
Δ*yecG*	0	21 ± 7	47 ± 12	10 ± 3	10 ± 10
Δ*ybdQ*	0	11 ± 7	48 ± 9	27 ± 8	4 ± 4

**Table 3:** Swarming motility assay.

Strains	Swarm distance (mm ± standard error)
wt	36.4 ± 3.0
Δ*ynaF*	36.4 ± 3.5
Δ*uspAB*	40.6 ± 1.2
Δ*ydaA*	36.2 ± 1.9
Δ*yecG*	36.8 ± 0.9
Δ*ybdQ*	39.8 ± 1.4
Δ*fliGHI*	44 ± 3.0
Δ*motA*	33 ± 3.0

### Transcriptional Induction of Universal Stress Protein Genes Under Growth in LBNS

**Figure 6:**
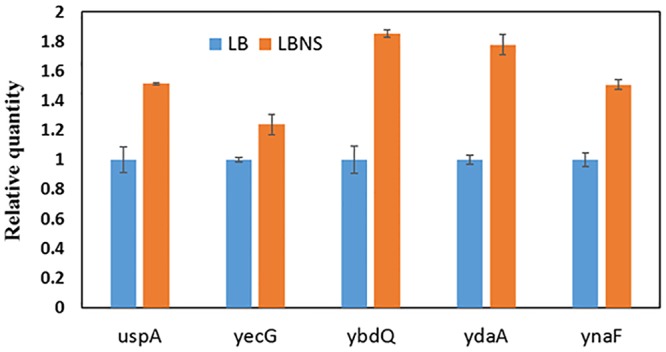
Relative quantification of gene expression in STm cells grown in LBNS (orange columns) compared to growth in LB broth (Blue columns) at 37°C. Gene expression was normalized to the expression of *rpoD* under the same growth conditions. Bars represent the standard deviation of three repeats. Each of the tested genes was significantly (*P* < 0.05) induced upon growth in LBNS compared to LB, according to unpaired Student’s *t*-test.

The data presented suggest that growth of STm under suboptimal conditions may induce the expression of the *usp*- and other genes that may facilitate leaf internalization. Since, the phenotypic analysis of the 5 *usp* genes was performed following growth of STm in LBNS, expression of the 5 *usp* genes was assessed following growth in LBNS compared to growth in LB at 37°C. Transcription of all the tested genes was induced following growth in LBNS compared to LB (Figure 6).

## Discussion

We have previously, demonstrated that *Salmonella enterica* cells cluster near lettuce stomata and penetrate through the stomatal opening into the inner leaf tissue in a process that involved chemotaxis (Kroupitski et al., 2009). The ability of HPOP to internalize leafy greens has been documented in many studies (Itoh et al., 1998; Seo and Frank, 1999; Takeuchi and Frank, 2001; Kroupitski et al., 2009; Golberg et al., 2011; Deering et al., 2012), however, it seems that internalization varies greatly in different studies and is influenced by genetic factors and environmental of both bacteria and plants (Mitra et al., 2009; Zhang et al., 2009; Deering et al., 2012; Wright et al., 2013).

In most studies, HPOP internalization was examined following growth in rich laboratory media (Itoh et al., 1998; Seo and Frank, 1999; Takeuchi and Frank, 2001; Solomon et al., 2002; Kroupitski et al., 2009; Niemira and Cooke, 2010; Golberg et al., 2011). In the present study, we have examined the idea that modulation of the growth conditions may affect STm internalization. We have demonstrated that leaf internalization was enhanced following pregrowth of STm under suboptimal growth conditions, representing bacterial adaptation to mild stresses, such as low salt (LBNS medium), O_2_-limitation, low nutrients (0.5×LB) and temperature (42°C). The obtained effect was not limited to STm, at least in regard to pregrowth in LBNS, as growth of *E. coli* O157:H7 in this medium has also improved leaf internalization compared to growth in LB broth. Internalization of *E. coli* O157:H7 in leafy vegetables is a controversial issue. Some researchers have reported that *E. coli* O157:H7 may reside within leaf tissue (Seo and Frank, 1999; Takeuchi and Frank, 2000, 2001; Erickson et al., 2010), while others failed to demonstrate *E. coli* O157:H7 internalization into lettuce leaves, regardless the type of lettuce, age of plant, or strain (Zhang et al., 2009; Deering et al., 2012). Similarly, Mitra et al. (2009) also reported lack of internalization of *E. coli* O157:H7 in intact spinach leaves. Previous studies in our laboratory with *E. coli* O157:H7 also failed to demonstrate substantial internalization using our lettuce leaf model, describe in this study (unpublished data). While, other plant and bacterial factors may account for these contradictory results, it is possible that difference in bacterial growth conditions, or exposure to other extrinsic factors, prior to the internalization assay, may have account for this variability in the internalization efficiency.

The detailed mechanisms through which growth conditions alter the capacity of bacteria to internalize leaves are not known. It is anticipated that pregrowth conditions may adapt bacteria to cope with the plant host. In the case of pregrowth in low NaCl medium, it was previously reported that the expression of curli, also known as thin aggregative fimbriae (tafi) is induced during growth of *Salmonella* and *E. coli* in low osmolarity medium, such as LBNS (Zogaj et al., 2001). Curli are bacterial adhesin that mediate attachment to various surfaces and contribute to biofilm formation on abiotic surfaces (Jain and Chen, 2007). Several studies have documented the involvement of curli also in the attachment of *Salmonella* and *E. coli* to plants (Barak et al., 2005; Jeter and Matthysse, 2005; Boyer et al., 2007; Macarisin et al., 2012; Yaron and Römling, 2014). Furthermore, curli were shown to enhance the transfer of *S*. Typhimurium from contaminated irrigation water to parsley and contributed to *Salmonella* plant internalization (Lapidot and Yaron, 2009). Thus, it is possible that curli may also influence STm internalization into lettuce leaves. Testing internalization of STm curli mutants should provide a better understanding regarding their potential role in lettuce leaf internalization.

Leaf surface is considered a hostile environment, where both phytopathogens and HPOP encounter multiple stresses, such as limited nutrients, UV irradiation, temperature fluctuations and desiccation (Brandl, 2006; Delaquis et al., 2007; Underwood et al., 2007; Heaton and Jones, 2008). It has been suggested that leaf internalization is a stress evasion strategy adapted by some phytopathogens and HPOP (Heaton and Jones, 2008). It might be hypothesized that pregrowing of *Salmonella* under non-optimal conditions may have induced a general stress response to overcome anticipated stresses, which among other features, induces mechanisms that contributes to leaf internalization. This idea is supported by the recent study of Fornefeld et al. (2017), who demonstrated that STm cells grown in lettuce-medium persist longer in soil microcosm compared to cells grown in LB broth (Fornefeld et al., 2017).

Adaptation of *Salmonella* to environmental conditions is mediated largely by overlapping regulatory systems that control the expression of numerous genes (Alvarez-Ordóñez et al., 2015). Universal stress proteins (USPs) are prevalent in all three domains of life. In bacteria, USP have a role in adaptation to several stresses, including oxidative stress, high temperature, low pH and hypoxia and are likely to contribute to the adaption of bacterial pathogens to the human host environment (Kvint et al., 2003; Liu et al., 2007; Persson et al., 2007; O’Connor and McClean, 2017). Knowledge regarding the role of USPs in phytopathogen-plant interaction is very limited (Ramachandran et al., 2014, transcriptomic base), and no data were reported regarding their potential role in human pathogen-plant interactions. Knockout mutants of STm in *uspAB, yecG, ydaA, ynaF, and ydaA*, encoding for UspAB, UspC, UspE, UspF, and UspG, respectively, were tested for their capacity to adhere to and internalize lettuce leaves. Mutations in *uspAB, ydaA, yecG*, and *ybdQ* significantly reduced STm internalization compared to the wt strain, while mutation in the *ynaF* gene showed no phenotype. Similarly, transduction of the mutations in *uspAB, ydaA, yecG*, and *ybdQ* back to the wt strain, resulted in a similar phenotype. It seems that expression of both *uspAB, ydaA, yecG*, and *ybdQ* is needed for leaf internalization.

To further examine if these genes are indeed induced during growth of STm in LBNS, RT-RT PCR analysis demonstrated that each of the studied USP genes was induced under growth in LBNS compared to growth in LB at 37°C. The finding that *ynaF* expression was also induced, although the phenotype of the *ynaF* deletion mutant was not affected, implies that not all genes induced under growth in LBNS are necessarily critical for leaf internalization.

Remarkably, the attachment of all the mutants to the leaf surface was comparable to that of the wt strain (data not shown), indicating that *uspAB, ydaA, yecG*, and *ybdQ* were not merely required for bacterial attachment.

It has been reported that in *S*. Typhimurium the *uspA* gene is induced by metabolic, oxidative, and temperature stresses, and that mutation in *uspA* gene leads to reduced stress tolerance (Liu et al., 2007). UspA contributed to the *in vivo* virulence of *S*. Typhimurium in mice and to survival within the host (Liu et al., 2007). Additionally, *Salmonella* Enteritidis *uspAB* mutant had a decreased ability to contaminate eggs and to persist in harmful environments, such as in the oviduct and eggs shell (Raspoet et al., 2011). It has been reported that *Salmonella* faces similar stress in both mammalian and plant hosts (Schikora et al., 2011; Barak and Schroeder, 2012; Goudeau et al., 2013; Wiedemann et al., 2015; Cox et al., 2018). Our findings, regarding the involvement of specific USPs in STm colonization of internal leaf tissue, further support the idea that bacterial adaptation to stresses may be advantageous to confront comparable stresses in both mammalian- and plant-hosts.

## Conclusion

Internalization of STm in lettuce leaf was affected by bacterial growth conditions. Exposure of the pathogen to mild stresses enhanced leaf internalization, possibly due to bacterial preadaptation, which also contributes to lettuce leaf internalization. The universal stress genes *uspAB, ydaA, yecG*, and *ybdQ*, but not *ynaF* gene, are required for lettuce leaf internalization. Further characterization of their role in STm internalization is needed in order to better understand how *Salmonella* and possibly other HPOP adapt to the hostile plant environment.

## Author Contributions

SS and YK designed the study and analyzed the results. YK, RP, and EB performed the experiments, except for RT-RT PCR. RG planned and performed the RT-RT PCR experiments, analyzed, and summarized the results. SS and YK wrote the manuscript.

## Conflict of Interest Statement

The authors declare that the research was conducted in the absence of any commercial or financial relationships that could be construed as a potential conflict of interest.
